# The complete chloroplast genome of *Sinosenecio globigerus* (C. C. Chang) B. Nordenstam (Asteraceae)

**DOI:** 10.1080/23802359.2024.2309262

**Published:** 2024-01-26

**Authors:** Yi Wang, Bin Hu, Jingyi Peng, Qiang Zhou

**Affiliations:** College of Biology and Environmental Sciences, Jishou University, Jishou, Hunan, China

**Keywords:** Asteraceae, *Sinosenecio globigerus*, chloroplast genome, phylogenetic reconstruction

## Abstract

The genus *Sinosenecio* B. Nordenstam is a group of perennial or sometimes annual or biennial herbs in the family Asteraceae. Here, we have successfully assembled and characterized the complete chloroplast (cp) genome of *S. globigerus*, which shows a typical quadratic structure with an overall GC content of 37.4%, comprising a pair of inverted repeat regions (IRs) of 24,848 bp, a large single-copy region (LSC) of 83,379 bp and a small single-copy region (SSC) of 18,180 bp. 133 genes were annotated, including 88 protein-coding genes, 37 tRNA genes and eight rRNA genes. Further nucleotide diversity analysis indicated that three genomic regions (*acc*D-*psa*I, *trn*K-*rps*16, and *ycf*1) exhibited sufficient variability and thus could be recommended as valuable barcodes for the delimitation and identification of *Sinosenecio* species. Phylogenetic reconstruction presented clear interspecific relationships within *Sinosenecio*, which were supported to some extent by cytology, morphology and geographic distributions. Our study will provide valuable and high-quality genetic information to further elucidate the diversified mechanisms in *Sinosenecio*.

## Introduction

The genus *Sinosenecio* B. Nordenstam is a group of perennial or sometimes annual or biennial herbs in the tribe Senecioneae of the family Asteraceae, containing about 47 species mainly distributed in the central and southwestern regions of China (Liu 2010; Liu and Yang 2011; 2012; Liu et al. 2019; Zou et al. 2020; Chen et al. 2022; Peng et al. 2022a; Su et al. 2023a, 2023b). Previous studies based on cytology and molecular DNA fragments have shown that *Sinosenecio* encompasses two species assemblages, namely the *Sinosenecio s.s.* group and the *S. oldhamianus* group. The former group has a base chromosome number of *x* = 30, with strictly polarized endothelial cell wall thickenings, while the latter group holds *x* = 24 (rarely *x* = 13), with polarized and radial thickenings (Liu and Yang 2011, 2012; Liu et al. 2019; Zou et al. 2020; Peng et al. 2022a). Chloroplast (cp) genome, as ideal molecular data, has been frequently applied to studies in the fields of plant taxonomy and phylogeny (Wanga et al. 2021). To date, only four cp genomes of *Sinosenecio* species have been reported (Xu et al. 2019; Zhou et al. 2021; Xie et al. 2022; Peng et al. 2022b), a limitation that undoubtedly hampers the consolidation of intrageneric relationships at the genomic level. Here, we characterize the complete cp genome of *S. globigerus* (C. C. Chang) B. Nord., 1978., which will provide new and high-quality genetic information to further elucidate the diversified mechanisms within *Sinosenecio*.

## Materials and methods

**Figure 1.**
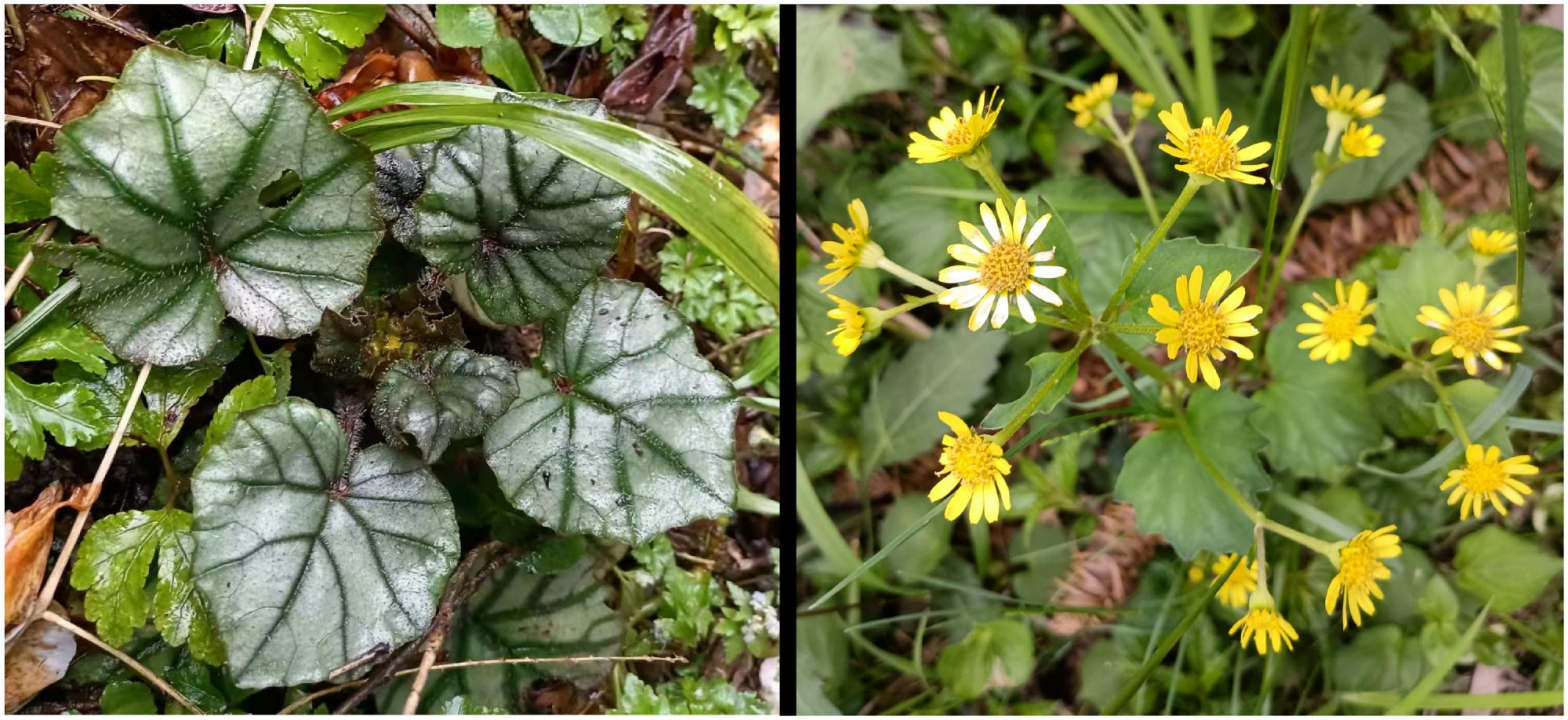
The living plant *Sinosenecio globigerus* was used in this study. This species is a stoloniferous herb, with leaf blades that are ovate-hairy, involucres without outer bracteoles, and ray and disk florets all lacking pappus. The photograph was taken by Xiao-Rong Lu in Badagongshan Mountain (Hunan, China), and was used with her permission.

*S. globigerus* (Figure 1) in this study was sampled from Badagongshan Mountain (29°37′11.06″ N, 110°9′50.41″ E), Hunan, China. Voucher specimen (JIU2023ZQ056) and DNA material were deposited at the herbarium of Jishou University (Hunan, China) (Qiang Zhou, zhouqiang@jsu.edu.cn). Total genomic DNA was isolated from the silica-dried leaf by using Plant Genomic DNA Kit DP305 (Beijing, China). The genome was sequenced on the Illumina Hiseq platform (Biomarker Technologies Co. Ltd., Beijing, China). In total, 10.3 G of the clean data were assembled into the cp genome sequence using GetOrganelle (Jin et al. 2018). The coverage depth was measured by mapping reads onto the cp genome sequence using bowtie2 to validate the correctness of assembly (Langmead and Salzberg 2012). The genome sequence was annotated on the website Geseq (Tillich et al. 2017), and annotated genes were manually checked and corrected for start and stop codon positions in Geneious v.9.0.2 with *S. baojingensis* Y. Liu & Q. E. Yang (Genbank: MZ325394) as a reference. The ultimate complete cp genome of *S. globigerus* was deposited in the GenBank database under the accession number OR752442. Additionally, The Chloroplot (Zheng et al. 2020) and Chloroplast Genome Viewer (CPGView) (Liu et al. 2023) were used to draw a circular map of the cp genome and splicing genes, respectively. For nucleotide diversity analysis, five available cp genome sequences of *Sinosenecio* species were aligned *via* MAFFT. A sliding window analysis of a window length of 600 bp and a step size of 200 bp was used in DnaSP v.6.12.03 to estimate the nucleotide diversity values (Rozas et al. 2017). We performed phylogenetic reconstruction by referring to Liu et al. (2023). 30 cp genome sequences were aligned using MAFFT, of which 28 species belong to the tribe Senecioneae and two to the tribe Anthemideae. The Maximum Likelihood (ML) analysis was performed using IQ-TREE with 1000 bootstrap replicates (Nguyen et al. 2015), and *Chrysanthemum indicum* Linnaeus and *Soliva sessilis* Ruiz & Pav. were chosen as outgroups.

## Results

**Figure 2.**
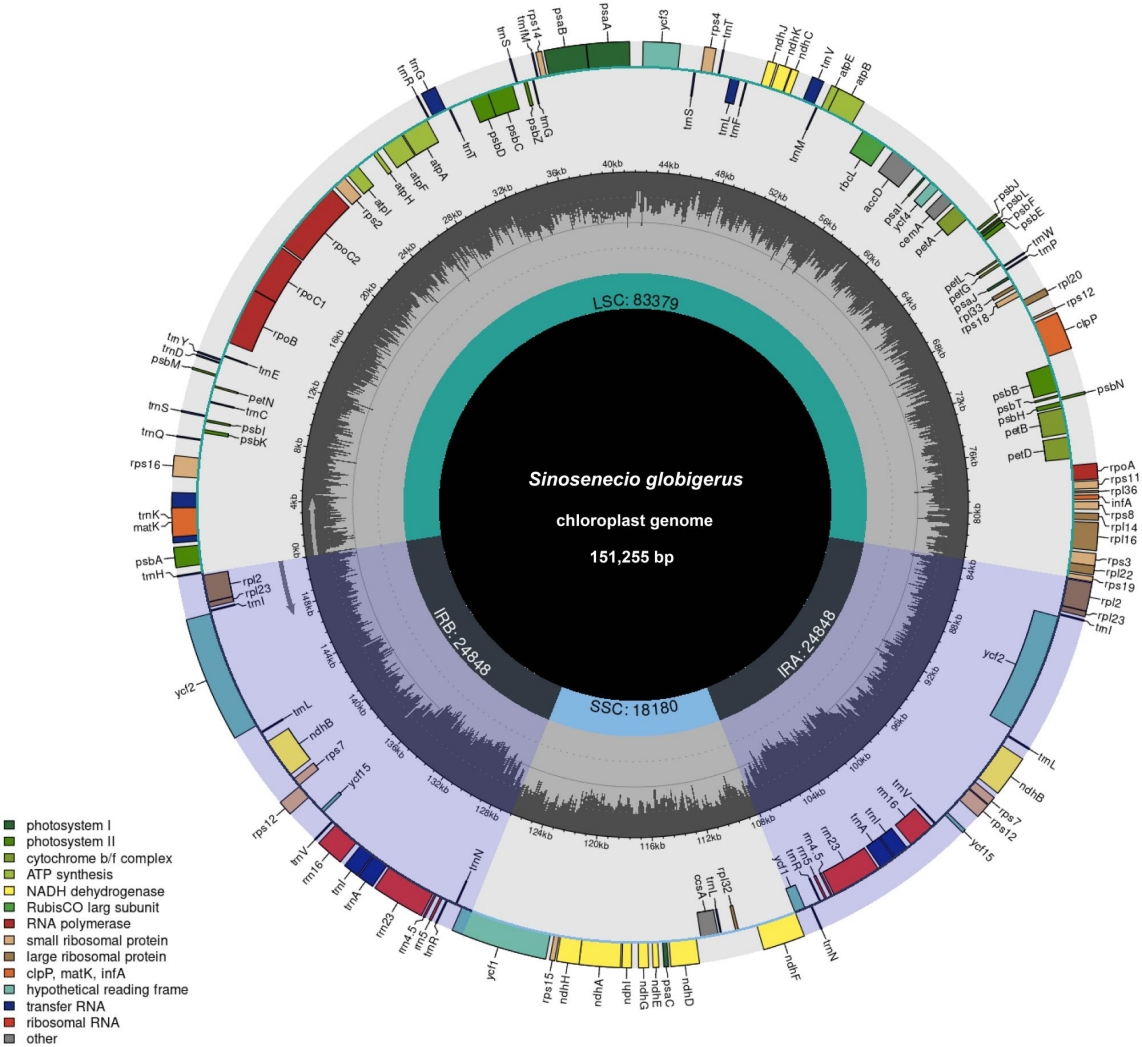
The gene map of the complete cp genome of *Sinosenecio globigerus* was drawn using the Chloroplot, showing the clockwise (genes inside the circle) and counterclockwise (outside) transcribed genes. Genes belonging to different functional groups are marked with different colors. In the inner circle, the darker gray area corresponds to GC content, and the lighter gray represents the at content.

**Figure 3.**
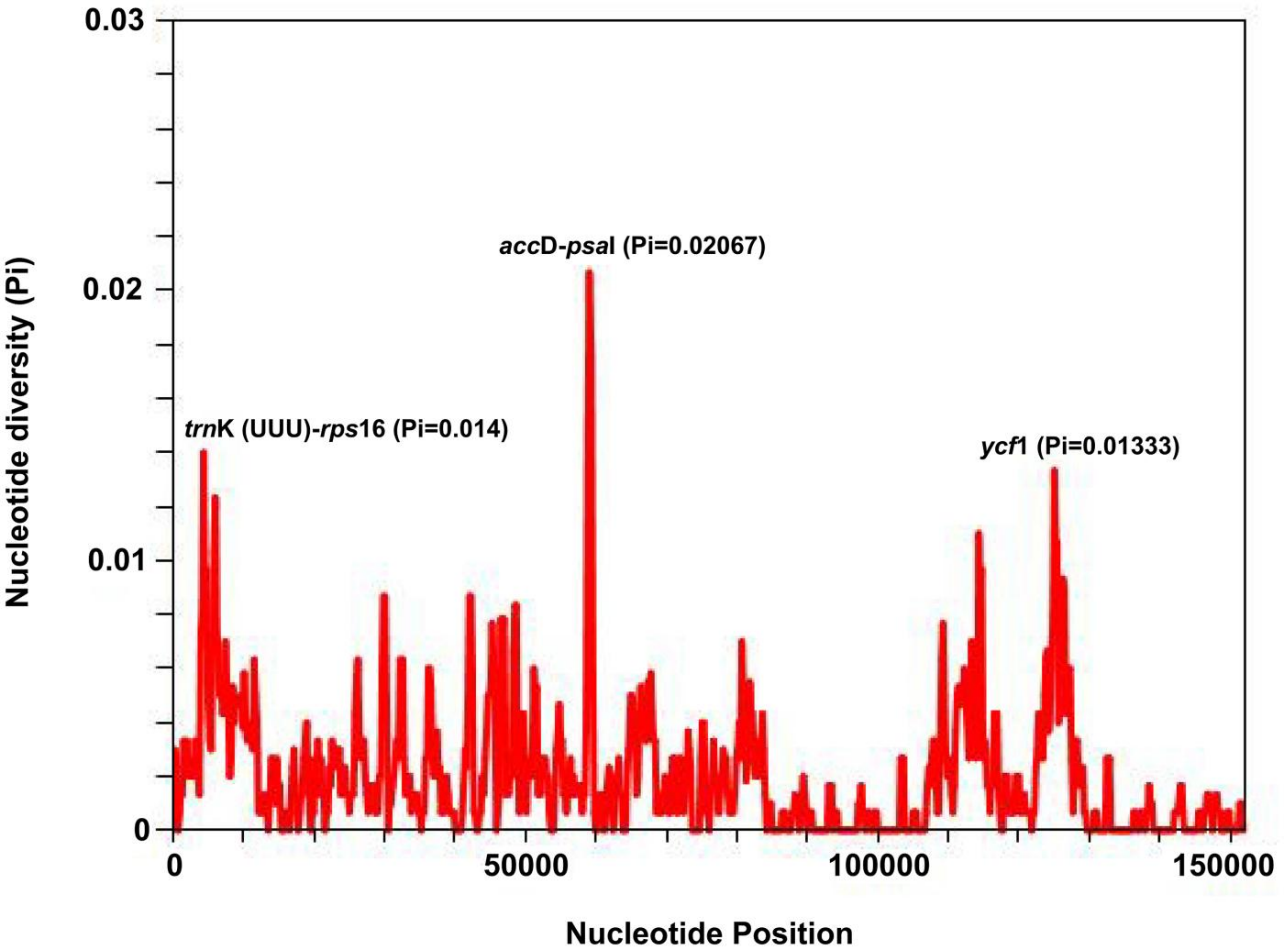
Sliding window analyses based on five cp genomes of *Sinosenecio* species using a window length of 600 bp and step size of 200 bp. The nucleotide diversity (Pi) value of each window is shown on Y-axis, and positions are shown on X-axis.

The cp genome of *S. globigerus* (Figure 2) is 151,255 bp in total size with an average coverage of 3018.91× (Supplementary material, Figure S1), showing a typical quadratic structure with a pair of inverted repeat regions (IRs) of 24,848 bp separated by a large single-copy region (LSC) of 83,379 bp and a small single-copy region (SSC) of 18,180 bp. A total of 133 genes were annotated, containing 88 protein-coding genes, 37 tRNA genes and 8 rRNA genes. Among these annotated genes, 11 cis-splicing genes including *rps*16, *atp*F, *rpo*C1, *ycf*3, *clp*P, *pet*B, *pet*D, *rpl*16, *rpl*2, *ndh*A and *ndh*B, and one trans-splicing gene *rps*12 were detected (Supplementary material, Figure S2). Three genes, *rps*19, *ycf*1 and *ndh*F were observed to span the regional boundary. Besides, the overall GC content of the cp genome is 37.4%, with corresponding values of 43.0%, 35.5% and 30.6% in the IR, LSC and SSC regions, respectively. Diving into genome divergence hotspots, nucleotide diversity analysis indicated that the Pi value ranged from 0 to 0.02779, with three hotspots showing relatively strong divergence (Figure 3). The intergenic spacer *acc*D-*psa*I manifested the apex divergence (Pi = 0.02067), followed by *trn*K (UUU)-*rps*16 (0.014), and *ycf*1 (0.01333), which were ranked as subsidiary divergence loci. Notably, the *ycf1* gene coding region displayed the highest degree of divergence among all other coding regions.

**Figure 4.**
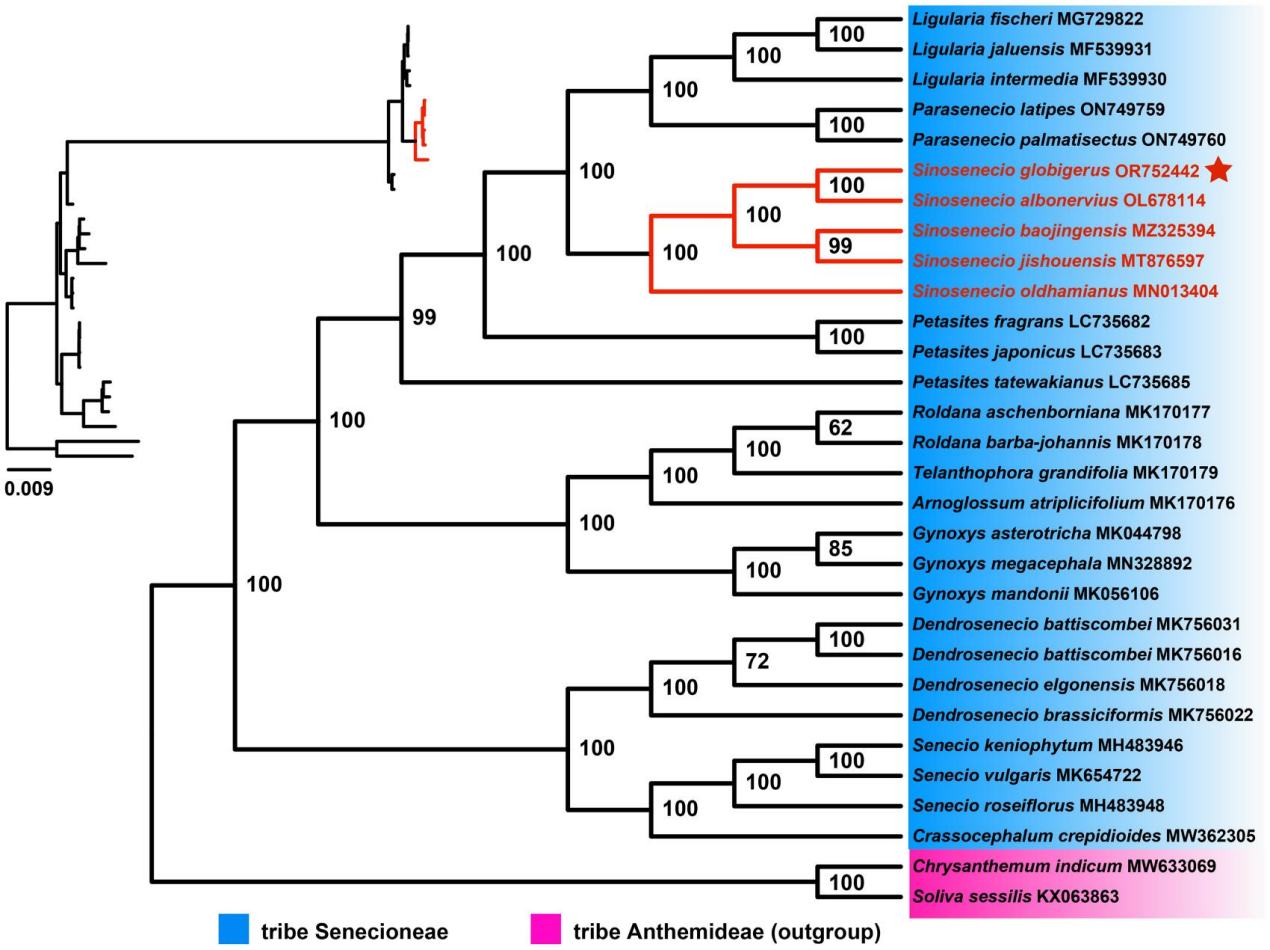
The phylogenetic tree with bootstrap support (BS) values was inferred from the ML analysis based on 30 cp genome sequences. The blue module and purple module represent the tribe Senecioneae and tribe Anthemideae (outgroup), respectively. The whole phylogenetic clade of *Sinosenecio* is marked with red, and the phylogenetic position of *Sinosenecio globigerus* is highlighted by the red star. Genbank number is given after each species name. The cp genome sequences used for phylogenetic analysis were all referenced and derived from Liu et al. (2023) study, as follows: *Ligularia fischeri* 729822, *Ligularia jaluensis* MF539931, *Ligularia intermedia* MF539930, *Parasenecio latipes* ON749759, *Parasenecio palmatisectus* ON749760, *Sinosenecio globigerus* OR752442, *Sinosenecio albonervius* OL678114, *Sinosenecio baojingensis* MZ325394 *Sinosenecio jishouensis* MT876597, *Sinosenecio oldhamianus* MN013404, *Petasites fragrans* LC735682, *Petasites japonicus* LC735683, *Petasites tatewakianus* LC735685, *Roldana aschenborniana* MK170177, *Roldana barba-johannis* MK170178, *telanthophora grandifolia* MK170179, *arnoglossum atriplicifolium* MK170176, *Gynoxys asterotricha* MK044798, *Gynoxys megacephala* MN328892, *Gynoxys mandonii* MK056106, *Dendrosenecio battiscombei* MK756016/MK756031, *Dendrosenecio elgonensis* MK756018, *Dendrosenecio brassiciformis* MK756022, *Senecio keniophytum* MH483946, *Senecio vulgaris* MK654722, *Senecio roseiflorus* MH483948, *crassocephalum crepidioides* MW362305, *Chrysanthemum indicum* MW633069, *Soliva sessilis* KX063863.

The matrix of 30 cp genome sequences contained 161,364 bp, and the ML phylogenetic tree with great resolution was presented in Figure 4. All *Sinosenecio* species clustered into an independent clade (BS = 100), with its sister clade consisting of two genera, *Ligularia* Cass. and *Parasenecio* W. W. Sm. & J. Small. Within the *Sinosenecio* clade, *S. oldhamianus* (Maxim.) B. Nord. was the first to differentiate, whereas sister relationships were found between *S. jishouensis* D. G. Zhang and *S. baojingensis* Y. Liu & Q. E. Yang (BS = 99), as well as between *S. albonervius* Y. Liu & Q. E. Yang and *S. globigerus* (BS = 100).

## Discussion

We assembled and annotated the cp genome of *S. globigerus*, which is similar in structure to the cp genome of other *Sinosenecio* species (Xu et al. 2019; Zhou et al. 2021; Xie et al. 2022; Peng et al. 2022b), whereas the high degree of structural conservativeness is characteristic of cp genomes in most angiosperms (Cheng et al. 2017). Our detection of mutable genomic regions is key to advancing diagnostic DNA barcode development (Li et al. 2022). In the present study, *acc*D-*psa*I, *trn*K (UUU)-*rps*16, and *ycf*1 were detected with sufficiently variable potential loci, which can be recommended as valuable barcodes for delimitation and identification among *Sinosenecio* species. Besides, cp genomes have proven to be powerful tools for exploring phylogenetic relationships in many plant groups, and we utilized them to reconstruct the phylogeny of the genus *Sinosenecio*. Inference from the ML analysis, *S. oldhamianus* position is located at the most basal part of the *Sinosenecio* clade, and *S. jishouensis*, *S. baojingensis*, *S. albonervius*, and *S. globigerus* form a subclade with more close relationships. Such a topology is essentially consistent with the one yielded based on nrITS (Zou et al. 2020). According to previous studies, these five species were defined as members of the *S. oldhamianus* group in *Sinosenecio*. Cytologically, the basal chromosome number of *S. oldhamianus* is *x* = 13, while that of the other four species is *x* = 24. Morphologically, *S. baojingensis* (in all florets) and *S. oldhamianus* (only in disk floret) have pappus, which is entirely absent in *S. jishouensis*, *S. albonervius* and *S. globigerus*. Geographically, *S. oldhamianus* is widely distributed, and for the other four species, the centers of diversity of their distributions overlap (NW Hunan and SW Hubei in China) (Liu 2010; Zou et al. 2020; Peng et al. 2022a). Although the present study recovered relationships among five *Sinosenecio* species, more organelle genomes need to be mined to consolidate the phylogenetic framework of *Sinosenecio* in the future.

## Conclusion

In this study, we characterized in detail the cp genome of *S. globigerus*. Phylogenetic reconstruction presented clear interspecific relationships within *Sinosenecio*, which were supported to some extent by cytology, morphology and geographic distributions. Our study will provide valuable genetic information for understanding the diversified formation of *Sinosenecio*.

## Supplementary Material

Supplemental Material

Supplemental Material

Supplemental Material

## Data Availability

The genome sequence data that support the findings of this study are openly available in GenBank of NCBI at https://www.ncbi.nlm.nih.gov/ under the GenBank accession OR752442. The associated BioProject, SRA and BioSample numbers are PRJNA1039893, SRR26830697 and SAMN38235998, respectively.
