# Supplementary material for: The complete chloroplast genome of Sinosenecio globigerus (C. C. Chang) B. Nordenstam (Asteraceae)
Source: Mitochondrial DNA B Resour. 2024 Jan 26;9(1):204–8. doi: 10.1080/23802359.2024.2309262 (PMC10823894; doi:10.1080/23802359.2024.2309262)
Supplement: Supplemental Material [file TMDN_A_2309262_SM4267.docx]

**The complete chloroplast genome of *Sinosenecio globigerus*(C. C. Chang) B. Nordenstam (Asteraceae)**

Yi Wang^1^, Bin Hu^1^, Jingyi Peng^1^, Qiang Zhou^1*^

1. College of Biology and Environmental Sciences, Jishou University, Jishou, Hunan, China

**Correspondence:** Qiang Zhou, zhouqiang@jsu.edu.cn

**Content:**

Figure S1. The coverage depth figure of the cp genome of *Sinosenecio globigerus*. The horizontal coordinate is the base of the plastid genome, and the vertical coordinate is the depth of sequencing corresponding to that base.

Figure S2. The schematic map of the cis-splicing (*rps*16, *atp*F, *rpo*C1, *ycf*3, *clp*P, *pet*B, *pet*D, *rpl*16, *rpl*2, *ndh*A and *ndh*B) and the trans-splicing (*rps*12) genes in the cp genome of *Sinosenecio globigerus*.


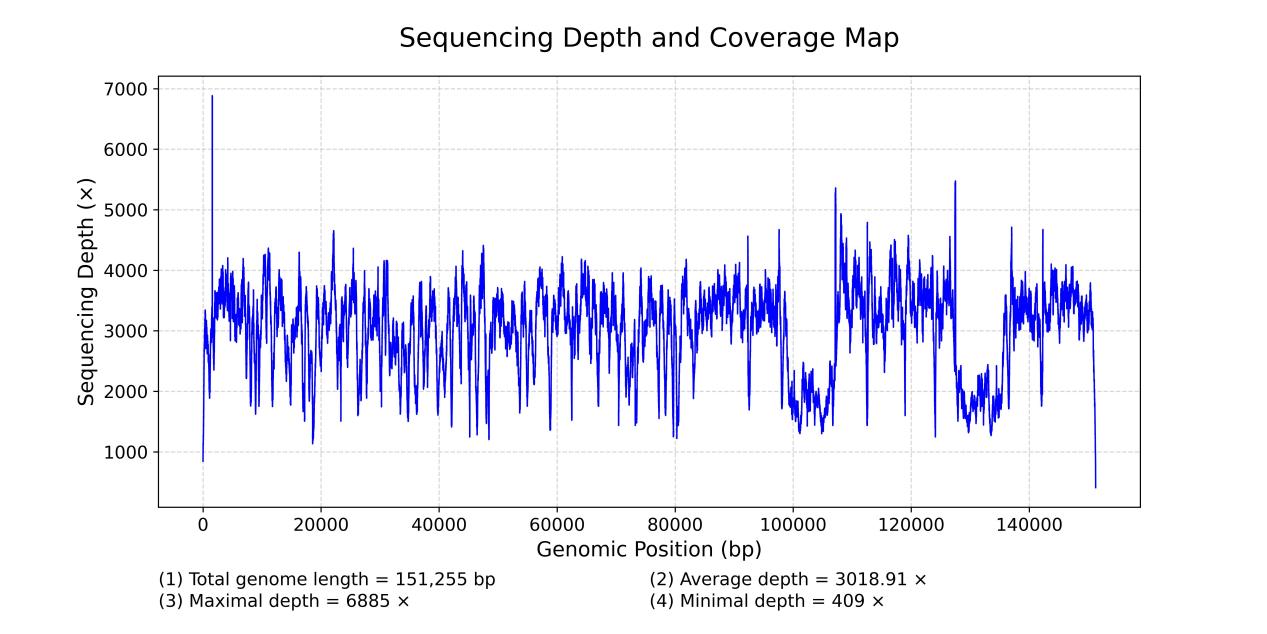


Figure S1. The coverage depth figure of the cp genome of *Sinosenecio globigerus*. The horizontal coordinate is the base of the plastid genome, and the vertical coordinate is the depth of sequencing corresponding to that base.


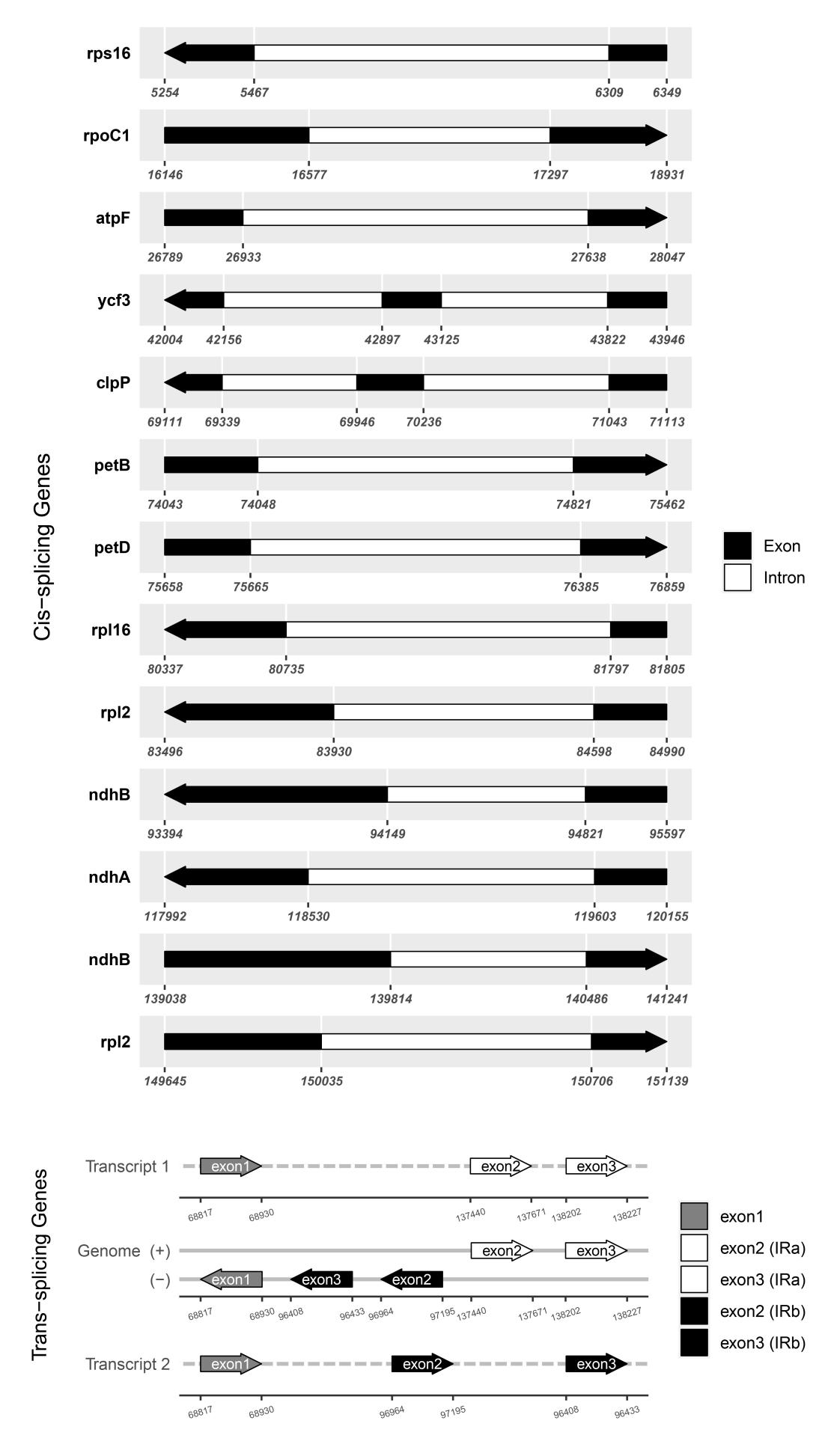


Figure S2. The schematic map of the cis-splicing (*rps*16, *atp*F, *rpo*C1, *ycf*3, *clp*P, *pet*B, *pet*D, *rpl*16, *rpl*2, *ndh*A and *ndh*B) and the trans-splicing (*rps*12) genes in the cp genome of *Sinosenecio globigerus*.
