# Supplementary figures and images for: The complete chloroplast genome of Sinosenecio globigerus (C. C. Chang) B. Nordenstam (Asteraceae)
Source: Mitochondrial DNA B Resour. 2024 Jan 26;9(1):204–8. doi: 10.1080/23802359.2024.2309262 (PMC10823894; doi:10.1080/23802359.2024.2309262)

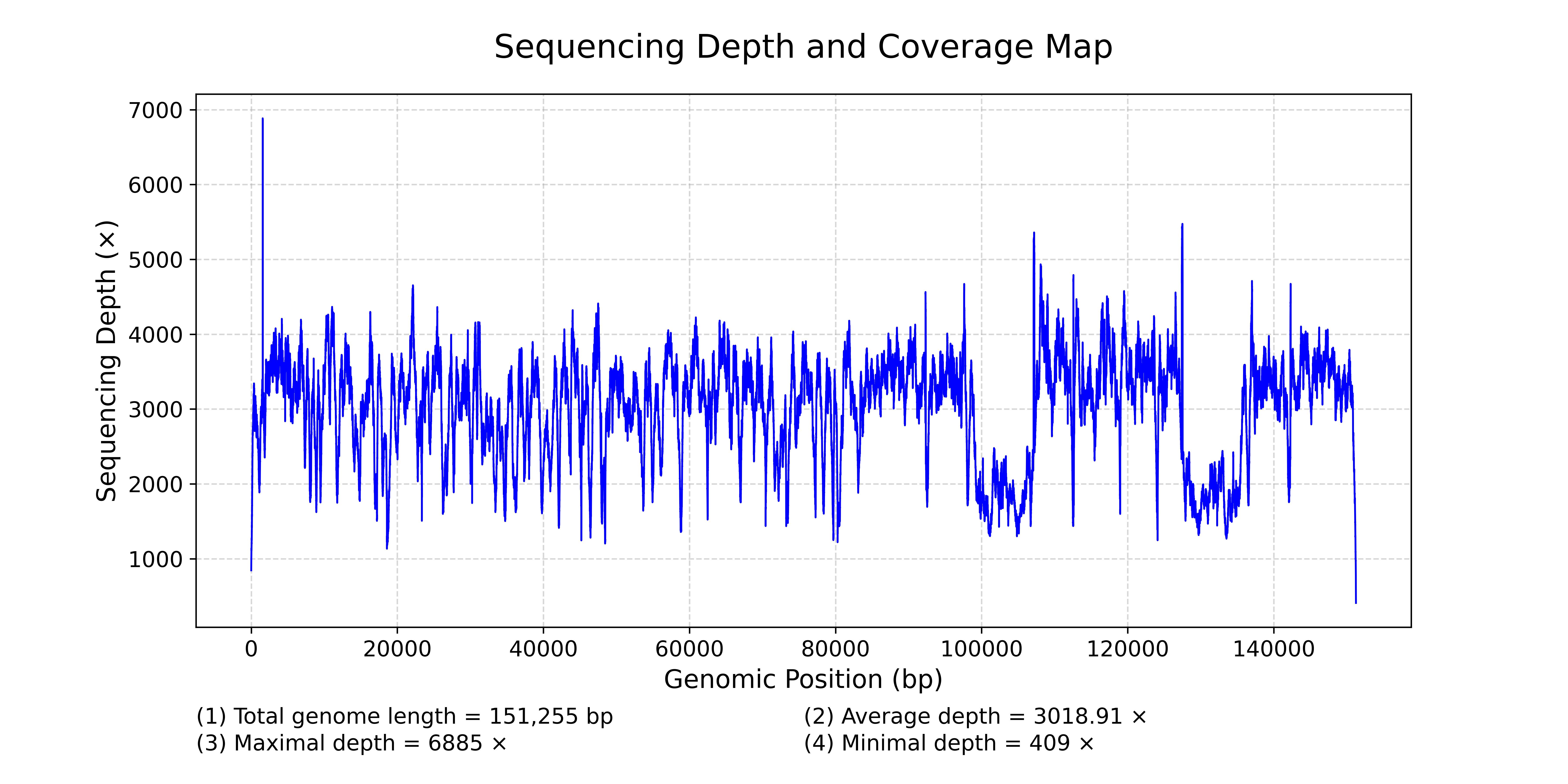

Supplement: Supplemental Material [file TMDN_A_2309262_SM4264.jpg]

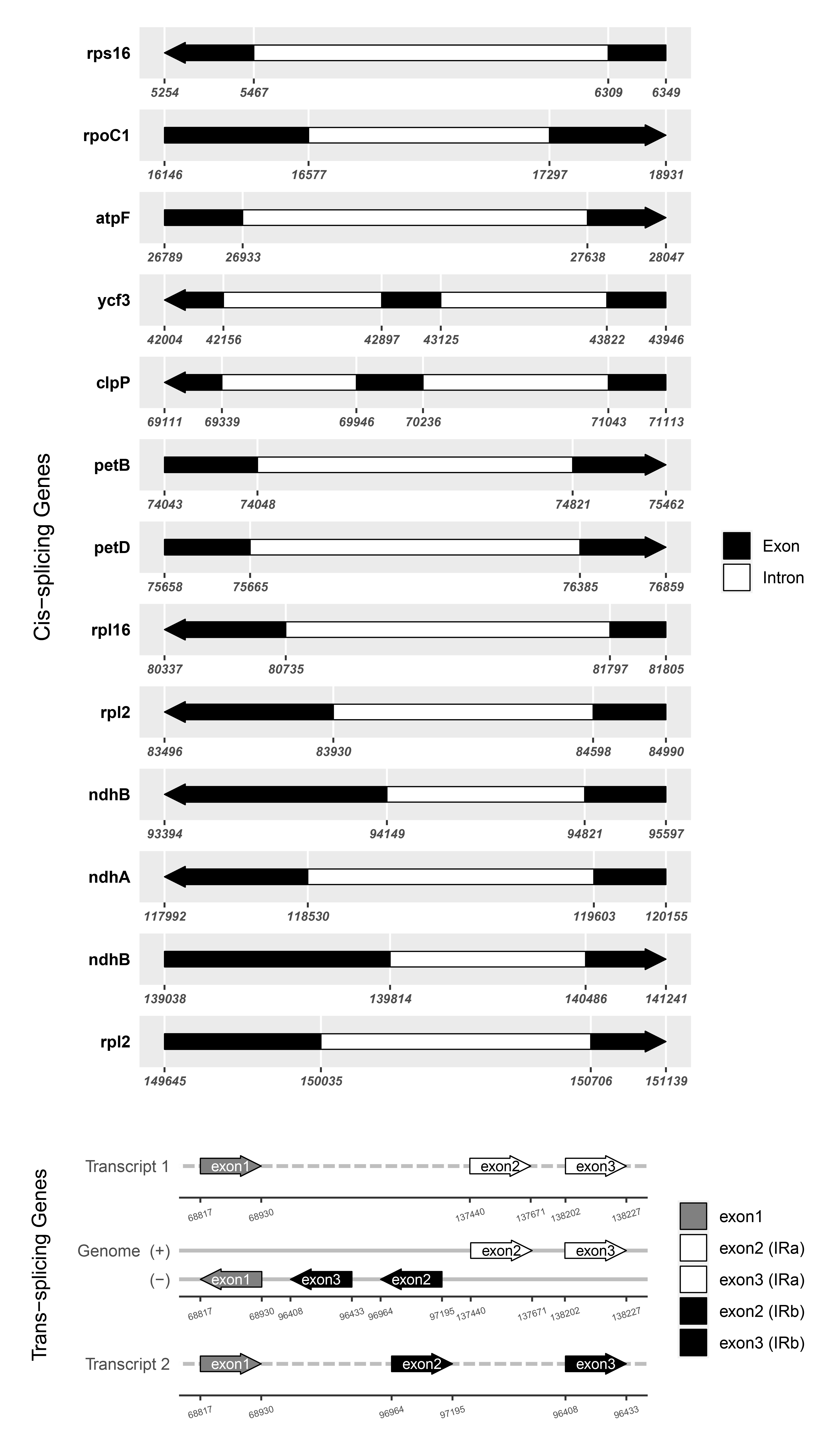

Supplement: Supplemental Material [file TMDN_A_2309262_SM4262.jpg]
